# The Impact of Tai Chi on Balance, Falls and Cognitive and Motor Health in Older Adults.

**DOI:** 10.1093/geroni/igaf122.1831

**Published:** 2025-12-31

**Authors:** Peter Wayne

**Affiliations:** Brigham and Women’s Hospital, Boston, Massachusetts, United States

## Abstract

As a mind-body exercise, Tai Chi has the potential to impact both cognitive and motor function. This presentation will highlight recent research regarding the effectiveness of Tai Chi in improving outcomes related to cognition, gait, balance and fall prevention. Drawing on both randomized trials cross-sectional studies with seasoned tai Chi experts, emphasis will be given to outcomes assessed under experimental dual task conditions that inform cognitive-motor interdependence.

